# Evaluation of nutritional value, characteristics, functional properties of *Cymodocea nodosa* and its benefits on health diseases

**DOI:** 10.1186/s12944-017-0626-z

**Published:** 2017-12-08

**Authors:** Rihab Ben Abdallah Kolsi, Hichem Ben Salah, Saber Abdelkader Saidi, Noureddine Allouche, Hafedh Belghith, Karima Belghith

**Affiliations:** 1 Laboratory of Plant Biotechnology Applied to the Improvement of Cultures, Faculty of Sciences of Sfax, 3038 Sfax, Tunisia; 2 Laboratory of chemistry of Natural Substances, Faculty of Sciences of Sfax, PB 802, 3018 Sfax, Tunisia; 3 Laboratory of ecophysiology, Faculty of Sciences of Sfax, 3038 Sfax, Tunisia; 4 Enzyme and Bioconversion Unit, Biotechnology Center of Sfax, University of Sfax, PB 1177, 3018 Sfax, Tunisia

**Keywords:** *Cymodocea Nodosa*, Nutritional value, LC–ESI–ms/ms, Biological properties

## Abstract

**Background:**

Nutritional fact study has prime importance to make the species edible and commercially viable to the food consumers. This is the first report that indicates the chemical characterization, functional, antioxidant and antihypertensive properties of *Cymodocea nodosa* to evaluate its nutritional status.

**Methods:**

Physico-chemical determination was determined by colorimetric and spectroscopic analysis. The functional and texture properties were evaluated since a desirable texture should be retained. Bioactive substances were determined by liquid chromatography-high resolution electrospray ionization mass spectrometry HPLC-DAD-ESI/MS2 analysis. Health benefit of this plant was highlighting by the antioxidant and antihypertensive potentials.

**Results:**

Results showed that the seagrass powder was characterized by a high content of fibers (56.4%), the fatty acids profile was dominated by the oleic acid, which represents about 62.0% of the total fatty acids and the functional properties proved important values of swelling capacity (6.71 ± 0.2) and water holding capacity (12.26 ± 0.25), that were comparable to those of some foodstuffs. Finally, the physico-chemical analysis shows the wealth in phenolic compounds, that could be explained by the high antioxidant and antihypertensive ability which was concentration dependent.

**Conclusion:**

The results from this study suggested that this marine plant could be utilized as a healthy food item for human consumption.

## Background

In recent years, the life style has given rise to important changes in our food habits, that’s why research has focused on the identification of the nutritional value of our aliments.

In Tunisia, as in all countries of the Maghreb and developing countries, the use of herbal medicine is widespread, and several herbal remedies used individually or in combination are recommended to treat a lot of diseases, obesity and hypertension in the population. As a consequence, nowadays, there is a huge interest among consumers and food industry on products that can promote health and well-being. These foods have been generically named functional foods, which were initially enriched with vitamins and/or minerals, such as vitamin C, vitamin E, folic acid, zinc, iron, and calcium [1]. Later, the approach changed to enrich foods with several micronutrients such as omega-3 and omega-6 fatty acids, Phyto sterols, soluble fiber (inulin and fructo-oligosaccharides, called prebiotics), etc., trying to promote consumers health or to prevent different diseases [1]. Seagrasses can be a very interesting natural source of new compounds with biological activity that could be used as functional ingredients. In fact, seagrasses are submerged marine angiosperms, producing flowers, fruits and seeds and they have separate roots, leaves and underground stems (rhizomes), which enable them to form an extensive network below the surface of water (Tropical topics, 1993). Human consumption of seagrasses is not entirely confined to the past. In South–East Asia, seeds of *Enhalus acoroides* are still a food source for coastal populations [2], whereas the rhizomes of *Cymodocea* spp. are used in the preparation of salad.

Studies on herbal plants, vegetables, and fruits have indicated the presence of antioxidants such as phenolics, flavonoids, tannins, and proanthocyanidins. Antioxidant agents of natural origin have attracted special interest because of their free radical scavenging abilities.

Along the Tunisian Mediterranean coasts *Cymodocea nodosa* (*C. nodosa*) is very frequent but not very studied, there are very few literatures involving the nutritional value of this species. It is most often simple signals herbarium, sometimes accompanied by brief descriptions of their structure and / or study of their associated fauna.

For these reasons, we proposed to develop this research in order to see the potential for valorization as a new functional ingredient of natural origin of this marine plant (*C. nodosa*) collected from Chebba (Tunisia).

## Methods

### Plant material

*C. nodosa* sample were collected from the coast of the Chebba in spring 2014, washed thoroughly with fresh water to remove epiphytes stored and other dirt particles, milled in a mechanical grinder for 5 min, to obtain a fine and homogeneous powder and then were stored in hermetic bags at room temperature (25°C).

### Physico-chemical analysis

#### Proximate composition

The dry matter was determined by drying a 1 g of sample in a thermo regulated incubator (Memert 40050ip20, Germany) at 105°C until constant weight [3]. Ash content was determined by incinerating the dry matter of the crude extract of the lyophilized polysaccharide in an electric muffle furnace maintained at 550°C [3]. Total sugars were determined according to the Dubois et al. method [4]. The proteins were assayed by the Bradford method [5]. The extraction of the lipids was carried out according to standard NF 03–713 [6]. The uronic acid value was quantified colorimetrically according to the method described by Blumenkrantz and Asboe-Hansen [7]. Total dietary fiber was evalueted by the gravimetric method proposed by AOAC [3]. A Hanna instrument 8418 pH meter (Switzerland) was used to measuring the pH at 20 °C. The water activity (aw) WAS measured at 25 °C using an apparatus (Novasina Aw Sprint TH-500, Switzerland).

#### Color parameters

The three-color parameters (L *, a *, b *) of the polysaccharide extract of *Cymodocea nodosa* were determined using a Minolta colorimeter Chroma Meter CR-300, CIE (1976) which is a color analyzer equipped with a measuring head. Calibration of the instrument is carried out before the analysis by placing the measuring head on a white plate.

In this CIE Lab coordinate system:

- The value of L * measures the brightness from 0 (black) to 100 (white),
- The value of a * ranges from −100 (green) to +100 (red),
- The value of b * ranges from −100 (blue) to +100 (yellow).

#### Chlorophylls and carotenoids contents

The total carotenoids content was determined according to the method of Britton [8].

Extraction of chlorophylls was realized by a modified method of Yoshii et al. [9] as follows: 0.5 g of the freeze-dried sample was mixed with 25 ml of ice-cooled acetone in the dark, the mixture stored at (−20 °C) in the dark for 18 h and the supernatant was filtered. Chlorophyll a and b contents were determined spectrophotometrically at 645 and 662 nm, respectively by the following equations:

$$ \mathrm{Chlorophyll}\ \mathrm{a}=11.75{\mathrm{A}}_{662}\hbox{--} 2.35{\mathrm{A}}_{645}\mathrm{Chlorophyll}\ \mathrm{b}=18.61{\mathrm{A}}_{645}\hbox{--} 3.96{0\mathrm{A}}_{662} $$

#### Mineral analysis

The main mineral contents (calcium, magnesium, potassium, sodium, zinc, copper and cadmium) in the *Cymodocea nodosa* polysaccharide extract are determined using an atomic absorption spectrophotometer (Hitachi Z6100, Japan) after acid attack by concentrated nitric acid (HNO_3_) [6].

#### Fatty acid composition

Lipids were extracted from the powdered *C. nodosa* by the method of Bligh and Dyer [10]. Esterification and analysis of fatty acid composition were carried out following the standard procedures (USP-NF, 2007b; PF, 2004). GC (ASHMACO, Model No. ABD20A Bio-Rad,Tokyo, Japan) was used for estimation of fatty acids. The gas chromatograph was equipped with a flame ionization detector maintained at 280 °C and a 50 m × 0.25 mm fused silica capillary column coated with macrogol 20,000 of thickness 0.25 μm. The injection port temperature was kept at 250 °C; the carrier gas was helium with the flow fate of 1 ml/min, the split ratio 1:100. Individual peaks of methyl esters were identified by comparison of the retention times and calculated equivalent chain length values with those of authentic standards.

### Functional properties of powdered seagrass

#### Water holding capacity (WHC)

The water absorption of *C. nodosa* sample was measured by the modified centrifugation method described by Gareau et al. [11]. The 3.0 g of sample were dispersed in 25 ml of distilled water and placed in pre-weighed centrifuge tubes. The dispersions were stirred and were left at 25 °C for 1 h, followed by centrifugation for 25 min at 3000 g. The supernatants were discarded and the moisture content of the samples was determined by dehydration in an oven for 25 min at 50 °C. The water absorption capacity was expressed as grams of water bound per gram of the sample on a dry basis.

#### Swelling capacity (SWC)

SWC of *C. nodosa* sample was analyzed by the bed volume technique after equilibrating in excess solvent. To 1.0 g of sample, in a 10 ml measuring cylinder, 10 ml of distilled water were added and the mixture was vigorously stirred. The measuring cylinder was left to stand for 18 h at room temperature. The swelling volume was measured and expressed as cm^3^ of swollen sample per gram of sample.

#### Oil holding capacity (OHC)

Samples (0.5 g) were mixed with 6 ml of corn oil in pre-weighed centrifuge tubes. The dispersions were stirred and left at 25 °C for 1 h, followed by centrifugation for 25 min at 3000 g. The oil supernatant was then removed and measured. The OHC of *C. nodosa* sample was expressed as the number of grams of oil held by 1 g of sample (DW) [11].

### DSC measurements

Thermal analysis of CNE was conducted with a Perkin–Elmer DSC4000. The sample (6 mg) was placed in aluminum pans, scanned from −50 °C to 250 °C at a rate of 5 °C/min, an empty aluminum pan was used as reference.

### Texture measurement

The texture properties of *C. nodosa* extract were determined by texture profile analysis (TPA test). A texture analyzer (TA.XT2; Stable Micro Systems, UK) was used to measure the force time curve for a two-cycle compression. The instrument provides two upward positive areas (1 and 2) and two downward negative curves areas (3 and 4). Areas 3 and 4 were observed just after the first compression (Area 1) and the second compression (Area 2), respectively. The TPA analyses were conducted using a cylindrical probe (16 mm diameter). The conditions were as follows: 15 mm compression, the test speed is of 0.5 mm/s, the posttest speed is of 1 mm/s and the time lapse between compressions was 20 s. All the operations were automatically controlled by the texture “Nexygen Lot” software connected to the instrument.

### Particle size distribution

The particle size distribution was determined by Laser scattering using a Malvern Master sizer 2000 (Malvern Instruments, Malvern, UK). Size distribution (10–20 g) sample was quantified as relative volume of particles in size bands presented as size distribution curves. The percentage of the particles having the same size was calculated by Software (Malvern Software V 2).

### Optical microscopy

Observations were carried out at room temperature using an optical microscope (Nikon Eclipse E400, Kanagawa, Japan) with a 40objective magnification. Some microgrammes of powder of the sample were placed on a glass slide and covered with a coverslip. The software employed for visualization was Lucia (version 4.5) and pictures were taken using a Basler video camera (Vision technologies, Ahrensburg, Germany).

### Preparation of hydroethanolic extract

The extraction was carried out on fresh green leaves of *C. nodosa*. Leaves were dried and powdered for the extraction. A mixture of ethanol and water (200 ml, 4:1 *v*/v) was added to the 50 g of *C. nodosa* powder and the mixture was kept under agitation for 24 h. Subsequently, the solution was filtered using Whatman No^°^3 filter paper. The extract was concentrated by evaporation to dryness at 40 °C, and the residue obtained was stored in glass vials, at 4 °C in the dark until further use.

### Determination of total phenolics, flavonoids and condensed tannins

Total content of phenolic compounds of *C. nodosa* extract was determined by the Folin-Ciocalteau method [12] using gallic acid as standard. Total flavonoid content was determined according to the method of Dewanto et al. [13] with minor modifications, using quercetin as standard. Sample (20 μl) was mixed with 30 μl of 5% (*w*/*v*) NaNO_2_ solution for 6 min, before addition of 60 μl of freshly prepared 10% (w/v) AlCl_3_ 6H_2_O. After 5 min, 200 μl of 1 M NaOH and 690 μl of bidistilled water were added and the absorbance was determined at 510 nm. Results are expressed as mg quercetin equivalent (QE) per g extract. Condensed tannins were measured according to the vanillin assay described by Sun et al. [14] using catechin as standard.

### Liquid chromatography–electrospray ionization–tandem mass spectrometry (LC–ESI–MS/MS) analysis

A reverse-phase high-performance liquid chromatography technique was developed to identify and quantify the major compounds contained in the *C. nodosa* extract. Concentrations were calculated based on peak areas compared to those of external standards. The HPLC chromatograph was a Schimadzu apparatus equipped with a (LC-10ATvp) pump and a (SPD-10Avp) detector. The column was (4.6 mm × 250 mm) (Shim-pack, VP-ODS) and the temperature was maintained at 40 °C. The flow rate was 0.3 ml/min. The mobile phase used was 0.1% phosphoric acid in water (A) versus 70% acetonitrile in water (B) for a total running time of 40 min, and the gradient changed as follows: solvent B started at 20% and increased immediately to 50% in 30 min. After that, elution was conducted in the isocratic mode with 50% solvent B within 5 min. Finally, solvent B decreased to 20% until the end of running time.

### Antioxidant activities

Free radical scavenging activity was evaluated with the DPPH (1,1-diphenyl-2-picrylhydrazyl radical) assay. The antiradical capacity of the sample extract was estimated according to the procedure reported by Brand-Williams et al. [15]. The ABTS^•+^ assay was based on the method of Re et al. [16]. Total antioxidant activity was determined according to the method of Kumaran and Karunakaran. [17]. Reducing power was determined by the method of Yamaguchi et al. [18].

### Determination of ACE inhibition activity

ACE inhibitory activity was measured in triplicate as reported by Kolsi et al. [19]. The IC_50_ value, defined as the concentration of CNE (mg/ml) required to inhibit 50% of ACE activity was calculated for sample using non-linear regression from a plot of percentage ACE inhibition versus sample concentrations.

### Statistical analysis

Data were expressed by mean ± SD. Statistical analysis was carried out by analysis of variance (ANOVA) and by Student’s t-test. A *p* < 0.05 was considered to be statistically significant.

## Results and discussion

### Physico-chemical analysis

The findings from the approximate composition analysis presented in Table 1 revealed that *C. nodosa* had 70.3% of dry matter and 16.4% of Ash. Based on several other researches [20, 21], the variation in these contents could presumably be attributed to the species, the stabilization and collection period. It is evident that there were wide variations in chemical composition amongst the various aquatic plants.

**Table 1:** Physico-chemical characteristics of *Cymodocea nodosa* seagrass

Components	Values (% dry weight)
Dry matter	70.3 ± 2.46
Ash	16.4 ± 1.05
Proteins	7.21 ± 0.11
Lipids	4.54 ± 0.02
Neutral sugars	7.28 ± 0,41
Uronic acid	8,31 ± 0,9
Total dietary fiber	56.40 ± 0.4
Insoluble fiber	32.20 ± 0.7
Soluble fiber	24.20 ± 2.5
pH	6.58 ± 0.18
Water activity	0.44 ± 0.03
CIE color	
L*	40.85 ± 0.07
a*	1.62 ± 0.02
b*	21.60 ± 0.04

All values given are means of three determinations (Χ ± SD); SD: standard deviation

The protein content found in the present study was relatively average (7.21% dry weight). This amount was in conformity with the value reported by Torbatinejad et al. [22] on some marine plants, ranged from 4.4% to 7.3%. However, this quantity was found to be lower than that of other research (28.7% - 18.6%) [23], variations in the protein content can be attributed to species differences and seasonal periods.

*C. nodosa* sample is relatively low in lipid (4.54%). These results are comparably lower than those reported by Ghoniemy et al. [23] for *C. nodosa* (36.5%) and *Ruppia cirrhosa* (15%), but higher than those estimated by Yamamuro et al. [24] for some seagrasses collected along the coast of Thailand. The differences could be attributed to factors such as climate, geography of development of the sample and to the method used to extract oil [25].

Marine plants are known as an excellent source of vitamins and minerals, due to their high polysaccharide content, which could also imply a high level of soluble and insoluble dietary fiber [26]. As shown in Table 1, the total dietary fiber was 56.40% DW, which proves it was the most abundant component in this seagrass. In this study, it was demonstrated that *C. nodosa* contained both soluble (24.20% DW) and insoluble (32.20% DW) dietary fiber (Table 1), which were in accordance than the values determined in green seaweeds [21].

The uronic acid content of *C. nodosa* extract (8.31%) was similar to those previously obtained by Yaich et al. [21]. The presence of uronic acids reflects the occurrence of soluble polysaccharide and the purity levels of the extract [20]. *C. nodosa* also had a high pH (6.58), this could be attributed to their high content in organic acids such as citric and malic acids, which are important for sensory properties and preservation [27].

Color influences the acceptability of food products, is one of the aesthetic properties that determine the suitability of *C. nodosa* to the application for which it is intended. As shown in Table 1, *C. nodosa* powder is colored; the high L* value (40.85) indicated that it had a light color, and the a * and b * values are in the order of (1.62) and (21.60), respectively, reflecting the relatively dark color of the product. Consequently, the use of this material could affect the color of the final product.

The *C. nodosa* sample showed low water activity (0.44), which is known sufficiently low to protect it against microbial alterations. The lower water activity is necessary for a better stability of the product by limiting the quantity of free water necessary for chemical reactions and proliferation of the microorganisms.

### Pigments determination

As shows in Table 2, the amounts of total chlorophylls (T-Chl), Chl a, Chl b and total carotenoids in *C. nodosa* seagrass were 0.067, 0.038, 0.029 and 0.929 mg/g DW, respectively. The values revealed that significant quantities of pigments were detected in this marine species. The nutrient composition of growth medium has an important influence on the production of photosynthetic pigments compounds by marine species.

**Table 2:** Chlorophylls and carotenoids content of *Cymodocea nodosa* seagrass

Pigment	Amount of pigments (mg/g DW)
Chlorophyll a	0.038 ± 0.009
Chlorophyll b	0.029 ± 0.005
Total Chlorophyll	0.067 ± 0.097
Carotenoids	0.929 ± 0.006

All values given are means of three determinations (Χ ± SD); SD: standard deviation

Chemical constituents of pigment produced by *C. nodosa* were as a resulting to nutrient composition of growth medium. This pigment composition is similar to that other angiosperms (*Enhalus acoroides, Halophila ovalis, H.beccarii Cymodocea serrulata*) which indicated that there is a clear seasonal variation in pigments concentration by registering the minimum value during the monsoon season when the water column was more turbid and the light availability to the seagrasses was limited causing light stress which is considered to be the important parameter for decreasing chlorophylls content and other photosynthetic growth parameters of seagrasses [28].

In general, the pigments contents can be affected by several environmental factors largely influenced by the availability of the light and morphology of the seagrass leaves and the depth in which the plants are growing [28].

### Mineral analysis

**Fig. 1:**
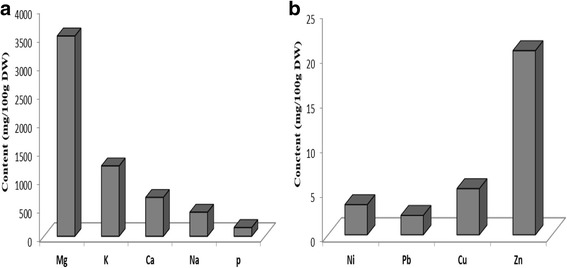
**a** Mineral composition of *Cymodocea nodosa* seagrass*.* **b** Heavy metals composition of *Cymodocea nodosa* seagrass

Marine plant are characterized by the presence of significant quantities of essential minerals. The composition of the *C. nodosa* in mineral elements (magnesium, calcium, potassium, sodium, phosphorus, zinc, plomb, nickel and copper) is illustrated in Fig. 1.

*C. nodosa* mineral fraction is composed mainly of magnesium (3510.17 mg/100 g dry weight), followed by potassium (1233.05 mg/100 g dry weight), while the other elements were Ca (680.55 mg/100 g dry weight), Na (421.285 mg/100 g dry weight) and P (152.965 mg/100 g dry weight). These remarkable levels of minerals can be attributed to the bioaccumulation capacity of the marine plant to nutrients in the environment where it survives [29]. These amounts can be attributed to the relative abundance of these elements in the surrounding water. Otherwise, it is difficult to compare the results of various investigations because of differences in treatments of the species and extraction procedures.

Several biological roles of many trace elements have been reported. Calcium and magnesium are essential trace elements in the structure and functioning of the human body. All of these properties influence their interactions with biological molecules of all natures. At the level of the physiological involvement of these two ions, one notes the importance of their equilibrium in the various compartments of the organism as well as their multiple functions in the different organs. Concerning the more specific study of these ions in terms of bone composition, renal function and transmission of nerve impulses, the observation of the different actions and regulations of calcium and magnesium makes it possible to highlight the complexity of their effects.

Heavy metals were regarded as being of the principal causes of environmental pollution, being given their toxicity, their persistence and their potential of bioaccumulation. The composition of the *C. nodosa* in heavy metals (zinc (Zn), copper (Cu), plumb (Pb), and nickel (Ni)) was shown in Fig. 1b. The content of seagrasses in this elements undergoes a wide range of variations with a trend in concentration of Zn > Cu > Ni > Pb in this species collected from the coast of Chebba. This was explained by the existence of higher levels of respiration and photosynthesis during the season of collect, which would favor the assimilation of metals.

### Fatty acid composition

The fatty acid composition of *C. nodosa* seagrass oil is given in Table 3. In the studied sample, the most abundant fatty acid was oleic acid C18:1 (62.43%) followed by palmitic acid C16:0 and linoleic acid C18:2. Kharlamenko et al. [30] reported that the major fatty acids of *Zostera marina* seagrass were C18:2 (n-6), C18:3 (n-3) and C16:0 collected from a shallow, semi-enclosed inlet of the Sea of Japan. The occurence of the C18 is important in human nutrition and for fish, which are not able to synthesise them. Fish can, however, elongate and desaturate dietary C18 and C16 fatty acids [31], which occur in the investigated marine plant (Table 2) in relatively high levels. Variations in the fatty acid contents are attributable to both environmental and genetic differences [25], mainly dependent on the local water temperature from which the seagrasses were collected with species containing higher levels of fatty acids with longer chain and higher degree of unsaturation compared to tropical species. Marine plants have a low lipid content compared to earth vegetables such as soy, sunflower, e.g. [25], thus being a low source of nutritional energy. Nevertheless, it is worth mentioning that the lipid fraction might contain higher levels of essential polyunsaturated fatty acids compared to other vegetables, which might be of interest if we consider the large amount and variety of seargasses.

**Table 3:** Fatty acid composition of *Cymodocea nodosa* seagrass

Fatty acid	Content (%)
C 16:0	21.65 ± 0.11
C 16:1	1.85 ± 0.05
C 18:0	5.09 ± 0.07
C 18:1	62.43 ± 0.17
C 18:2	7.56 ± 0.23
C 18:3	0.97 ± 0.03
C 20:0	0.07 ± 0.09
C 20:1	0.38 ± 0.02

All values given are means of three determinations (Χ ± SD); SD: standard deviation

### Techno-functional properties

The techno-functional properties of *C. nodosa*, such as the hydration properties that can be determined by swelling capacity (SWC) measurement, water holding (WHC) and oil holding (OHC) capacities was shown in Table 4, however, an important WHC of *C. nodosa* powder (12.26 g water/g DW) was noted. The water retention capacity is an important property for the ingredients in order to improve the organoleptic properties of the formulated product by limiting the phenomenon of syneresis and modifying the viscosity and texture of certain food products. In addition the SWC of this marine plant powder was (5.22 ml/g DW), it was relatively lower than the result obtained from the powder from some marine alga [32]. For the different marine species the differences in swelling index can be attributed to the different protein conformations and to the variations in the number and nature of the water binding sites. There is a strong correlation between water retention capacity and swelling capacity [33]. The OHC of the *C. nodosa* powder was described in Table 4 and the OHC value recorded was 1.63 g oil/g DW. This is another important property for the ingredients used in food formulations as well as for stabilizing emulsions and foods with a high fat content. This property is therefore exploited in food to improve their oil retention which is normally lost during cooking [34].

**Table 4:** Functional properties of *Cymodocea nodosa* seagrass

Properties	Values
Water holding capacity: WHC (g water/g DW)	12.26 ± 0.25
Swelling capacity: SWC (ml/g DW)	6.71 ± 0.2
Oil holding capacity: OHC (g oil/g DW)	1.63 ± 0.03

All values given are means of three determinations (Χ ± SD); SD: standard deviation

### Texture properties

Textural parameters of *C. nodosa* seagrass, such as firmness, springiness, cohesiveness, adhesiveness and chewiness are presented in Table 5. *C. nodosa* sample exhibited a highest firmness (1.9 N), the hardness (firmness) changed with the variety and the composition of sample; it was probably related to the sample structure.

**Table 5:** Texture properties of *Cymodocea nodosa* seagrass

Texture properties	Values
Firmness (N)	1.90
Springiness (mm)	11.60
Cohesiveness	0.58
Adhesiveness (N)	0.92
Chewiness (N mm)	6.83
Breaking force (N)	1.44
Rigidity (N/mm)	0.79
Adhesiveness force (N mm)	0.36
Thickness (mm)	23.00

All values given are means of three determinations (Χ ± SD); SD: standard deviation

Firmness is related to cohesiveness in that it is the force which is necessary to attain a given deformation; it describes the extent to which a material can be deformed before it ruptures. In this context, the sample presented a (0.58) cohesiveness value. Springiness means how well a product physically springs back after it has been deformed during the first compression. The springiness of *C. nodosa* (11.6 mm) was rather higher. Adhesiveness is an important parameter for food products, is more of a surface characteristic and depends on a combined effect of adhesive and cohesive forces, the result of sample adhesiveness value (0.92 N) was recorded in Table 5. The tested sample has an important resistance to chewing forces (6.83 N mm). Chewiness is the quantity to simulate the energy required for masticating a semi-solid sample to a steady state of swallowing. Indeed, *C. nodosa* marine plant presented highest firmness, springiness, cohesiveness and chewiness; these results are in line with some recent studies of algae and dietary fiber made by Ayadi et al. [35], yaich et al. [20], but it’s the first report that indicates the textural properties of *C. nodosa* species.

The peak force attained during the test is referred to as adhesiveness and breaking force. This can be explained by cells membrane deterioration during freezing inducing the loss of binding capacity among cell walls. As consequence *C. nodosa* lose her firmness (0.79 N/mm of rigidity) and reduce their thickness (23 mm).

### The morphology of *C. nodosa* extract

**Fig. 2:**
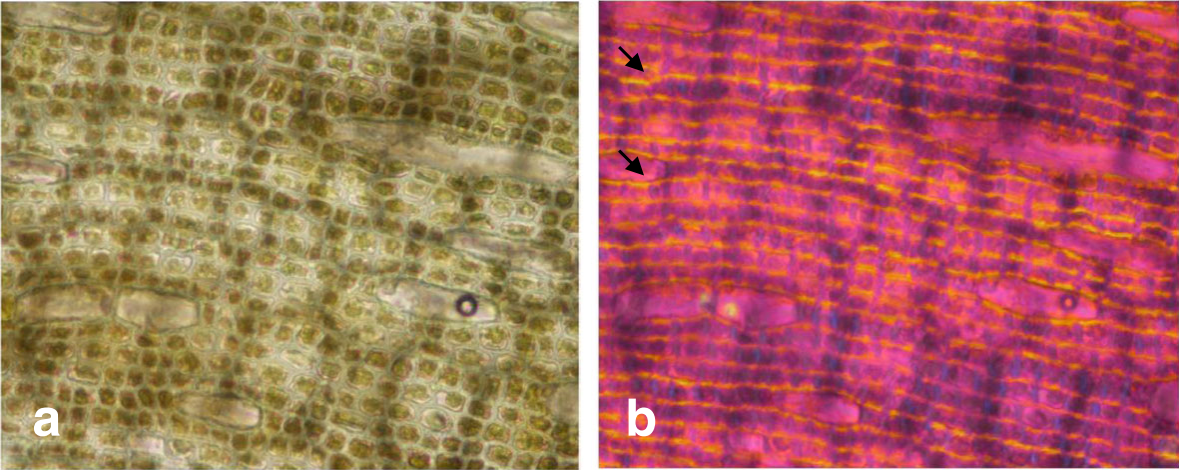
Micoscopy observation of *Cymodocea nodosa* in the natural state powder (**a**: analyzer mode; **b**: polarizer mode)

*C. nodosa* sample was first examined using an optical microscope provided with analyzer and polarizer. Fig. 2 (a) shows an example of a broken blade and fibers are clearly visible at the fractured end. These microscopic analyzes clearly reveal that this marine plant is rich on very fine fibers that are aligned horizontally with coloring green filaments of the plant. These appear to be approximately circular, with diameters between 4 and 5 μm. It can be seen that the eelgrass can be considered as a composite, with fibers reinforcing a matrix. The blades are organized in a closed cell structure; they are present in bundles of 7 to 10 fibers mainly located near the outer surfaces of the blade on the faces of this structure (Fig. 2 (a)).

Thus the optical microscope, provides macroscopic information on crystalline structure which corresponds to the areas of yellow and blue sky in Fig. 2b, that is optically anisotropic. This anisotropy allows for polarized light contrast depending on the orientation of the particles.

### Particle size distribution

**Fig. 3:**
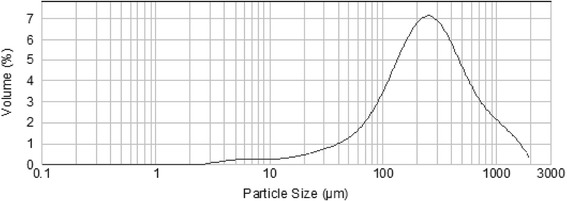
Particle size distribution of *Cymodocea nodosa* powder

Figure 3 shows particle size distributions of *C. nodosa* extract which are fell into two populations: a minority of particles was smaller than 0.1–1 μm, while the majority was larger than 10 μm. According to the Laser particle size analyzer, the particle size of *C. nodosa* powder could be divided into five fractions: 0.1–1, 1–10, 10–100, 100–1000, 1000–3000 μm. The highest percentage of dried sample was observed in the fourth fraction (100–1000). Whereas, the lowest percentage was in the first and the second fractions. Results showed that *C. nodosa* powder obtained had bulkier particles. This can be attributed to the improved green density, hence a higher number of contact points among the particles, and the higher fractions of fine particles hence shorter diffusion paths, in the resultant compact [36].

According to Guillon and Champ [37], the particle size distribution depends on the type of cell walls and on the technological treatments (degree of grinding, thermal treatment, etc.). Considering the highest fiber content observed in *C. nodosa,* it would be at the origin of this substantial difference size distribution powders.

### Thermal analysis

**Fig. 4:**
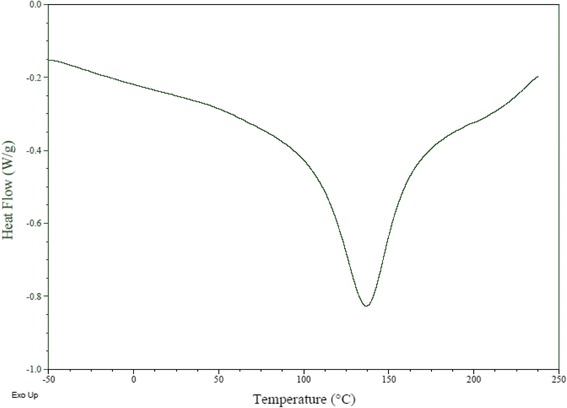
Thermal comportment by differential scanning calorimetry of *Cymodocea nodosa* seagrass

The differential scanning calorimetry (DSC) is the most used technique to analyze the characteristics of thermal comportment of samples. It has been used extensively as a tool in food research to study the thermodynamics and kinetic properties of sample behavior in solution and solid states. DSC thermograms (Fig. 4) presented the main thermal event between −50 °C and 250 °C, it was an endothermic peak around 150 °C that can be related to a glass transition temperature, due to the presence of proteins, fibers (pectin, lignin, hemicellulose and cellulose), and water in the *C. nodosa* sample. Indeed, it is well known that water has a negative glass transition temperature (−173 °C) as opposed to the different constituents of seeds that have a positive glass transition temperature (sucrose: 62 °C; pectin: 160 °C; Hemicellulose: 150–220 °C; cellulose 220–250 °C; protein: 77–112 °C) [38]. Which can be explained by the increase of the relative amount of fibers and the low amount of water in *C. nodosa* indicating that this is an advantage for a better conservation of sample.

### Phenolic profile using HPLC-DAD-ESI/MS^2^

**Fig. 5:**
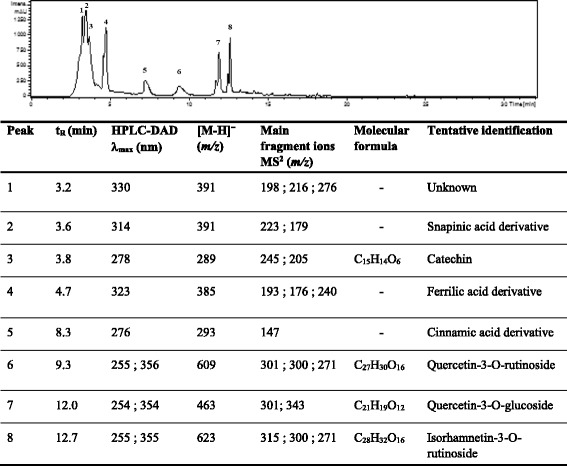
Identification of phenolic compounds of *Cymodocea nodosa* extract by HPLC-DAD-ESI/MS^2^

The phenolic constituents of the *C. nodosa* extract were analyzed for the first time. On the basis of the UV/DAD, MS and MS^2^ data, approximately eight phenolic compounds were detected and tentatively identified. The HPLC-UV/DAD chromatogram at 254 nm and the retention times (t_R_), UV values (λ_max_) and the molecular ions of the phenolic compounds are illustrated in Fig. 5.

In this study, four flavonoids (3, 6, 7 and 8) and three phenolic acid derivatives (2, 4 and 5) were characterized from the *C. nodosa* extract. Peak 1 (t_R_ 3.2 min; λ_max_ 330 nm) gave a molecular ion [M-H]^ˉ^at *m/z* 391. Its MS^2^ spectrum presented two fragment ions at *m/z* 216 and *m/z*198, however, the compound 1 could not be identified. Peak 2 (t_R_ 3.6 min; λ_max_ 314 nm) was tentatively identified as sinapic acid derivative according to the fragment ions at *m/z* 223 [sinapic acid-H] and at *m/z* 179 [sinapic acid-H-CO_2_] observed in MS^2^ spectrum.

Compound 3 displayed UV maximum absorption at 276 nm and gave [M-H]^ˉ^ ion at *m/z* 289. It was identified as catechin [39, 40]. Peak 4 (t_R_ 4.3 min; λ_max_ 323 nm) having [M-H]^ˉ^ion at *m/z* 385 and Peak 5 (t_R_ 5.2 min; λ_max_ 276 nm) with [M-H]^ˉ^ion at *m/z* 293 were tentatively assigned as ferulic and cinnamic acid derivatives, respectively. They presented characteristic MS^2^ ions at *m/z* 193 [ferulic acid-H] and at *m/z* 147 [cinnamic acid-H].

Concerning compounds 6, 7 and 8, their UV spectra exhibited two maximum absorptions in the 254-255 nm range and in the 354–356 nm range, characteristics of flavonols. Peaks 6 (t_R_ 8.3 min) and 7 (t_R_ 10.2 min) showed parent ions [M-H]^ˉ^ at *m/z* 609 and at *m/z* 463, respectively. They exhibited the same main MS^2^ ion at *m/z* 301 which corresponds to quercetin aglycone. Thus, they were characterized as quercetin-3-O-rutinoside and quercetin-3-O-glucoside [40]. The MS^2^ fragment ion at *m/z* 315 of compound 8 is assigned to isorhamnetin derivative. Moreover, this compound showed [M-H]^ˉ^ ion at *m/z* 623, therefore, it was identified as isorhamnetin-3-O-rutinoside [40].

### Determination of phenolic compounds

Given the diversified phenolic profile of *C. nodosa* extract, as described by the HPLC-DAD-ESI/MS^2^, the Follin-Ciocatleau, AlCl_3_ and vanillin colorimetric methods were performed to mesure total phenols, flavonoid and condensed tannins contents respectively. The obtained results showed that *C. nodosa* extract contains 122.22 ± 13.36 mg GAE/g extract, 81.5 ± 9.83 mg QE/g extract and 54.58 ± 13.65 mg CE/g extract of the assessed compounds respectively (Table 6).

**Table 6:** Content of total phenolics, flavonoids and condensed tannins of *Cymodocea nodosa* extract

Phenolic contents	Values
Total phenols (mg GAE/g extract)	122.22 ± 13.36
Flavonoids (mg QE/g extract)	81.50 ± 9.83
Condensed tannins (mg CE/g extract)	54.58 ± 13.65

All values given are means of three determinations (Χ ± SD); SD: standard deviation

This marine species contains active compounds at various qualitative and quantitative levels. Our results confirmed those reported by others who found that marine organisms produced large variety of secondary metabolites leading to the development of new bioactive compounds of various chemical classes [41]. For example, it was reported in this study that the *C. nodosa* extract possesses higher concentrations of total phenols and flavonoids compounds. Similar results were described by Pradheeba et al. [28], they emphasized that quantitative estimation of the leaves of eight seagrass species proved highest concentration of phenolic compounds, such higher phenol content in seagrass leaves could be attributed to the defense mechanism of the plant against the epiphytes.

Besides, condensed tannins are present in low concentrations (54.58 mg CE/g extract) compared to total phenols and flavonoids compounds. Similar results were described by other studies in the literature for other extracts of plants [42]. According to the literature, leaves may contain more antioxidant molecules than other parts of the plant. Phenolic compounds of plants have potent antioxidant activities and have been shown to be highly effective scavengers of most antioxidant molecules, including singlet oxygen, and various free radicals. So, comparable with the findings in the literature for other extracts of plant products [43], our results suggested that phenolic and flavonoids compounds may be the major contributors for the antioxidant activities.

### Antioxidant capacities

**Fig. 6:**
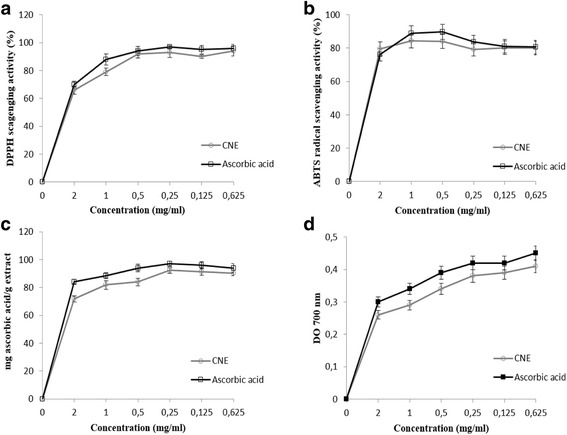
The in vitro antioxidant activities of CNE at different concentrations. DPPH free radical-scavenging activity (**a**), ABTS radical-scavenging activity (**b**), Total antioxidant activity (**c**) and reducing power (**d**)

The results of the in vitro antioxidant activities of *C. nodosa* seagrass are shown in Fig. 6. In the present study, the scavenging DPPH, ABTS, reducing power and total antioxidant ability of hydroethanolic extract was concentration dependent and it was comparable to the ascorbic acid. According to the literature, DPPH is one of the compounds that possessed a proton free radical with a characteristic absorption, which decreases significantly on exposure to proton radical scavengers. DPPH radical was scavenged by antioxidant through donation of hydrogen to form a stable DPPH molecule. The concentration of antioxidant needed to decrease the initial DPPH concentration by 50% (IC_50_) is a parameter widely used to measure antioxidant activity. As the IC_50_ value of the extract decreases, the free radical scavenging activity increases. In the present study, the investigated extract expressed the ability to scavenge the stable DPPH free radical reaching 50% of reduction with an IC_50_ value 0.33 mg/ml conformed to vitamin C. The same result was obtained using the ABTS radical, the decolonization of ABTS^•+^ cation radical is an unambiguous way to measure the antioxidant activity of phenolic compounds. The scavenging effect increased with concentrations. Indeed, the IC_50_ of CNE was evaluated at 0.40 mg/ml against 0.38 mg/ml for ascorbic acid as a positive control.

Our results are in agreement with those described for other extracts from five species of seagrasses and six species of seaweeds which were collected from the Gulf of Mannar, on the southeastern coast of India [44]. However, the CNE exhibited important values for the total antioxidant activity and the reducing power (90.36 mg/ml and 0.41, respectivily) very similar to the vitamin C (94 mg/ml and 0.45, respectivily). The reducing properties are generally associated with the presence of reductones, which have been shown to exert antioxidant effect by breaking the free radical chain by donating a hydrogen atom. Reductones are also reported to respond to various precursors of peroxides, thus, preventing their generation. The reducing capacity of a compound may serve as a significant indicator of its potential antioxidant capacity.

This antioxidant activity could be explained by the high content of polyphenolic, flavonoid in CNE. Diverse pharmacological activities inherent to medicinal plants have been attributed to their phenolic composition. The antioxidant capacity of phenolic compounds is principally due to their redox properties, which make them act as hydrogen donors, reducing agents, and singlet oxygen quenchers. The chemical composition and structures of active extract components are important factors governing the efficacy of natural antioxidants. Further studies are needed for the isolation and identification of individual phenolic compounds and the assessment of their antioxidant activities concerning the *C. nodosa* species.

### ACE inhibitory activity of CNE

One of the therapeutic approaches to hypertension is the use of angiotensin converting enzyme (ACE) inhibitors. Thus, the renin-angiotensin system plays a pivotal role in the regulation of blood pressure, electrolytes and blood volume, and in the pathophysiology of cardiovascular diseases. Since the synthetic ACE inhibitors may cause adverse side effects, plant molecules could be used as natural and economical ACE inhibitors for hypertension prevention.

**Fig. 7:**
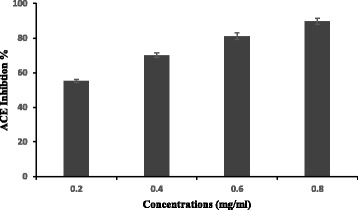
ACE-inhibitory activity of *Cymodocea nodosa* extract at different concentrations

In this context, we were interested in the in vitro study of the effect of CNE on ACE inhibitory activity (Fig. 7). The results revealed that the inhibitory activity of CNE was concentration dependent. The highest ACE inhibitory activity (89.76 ± 1.75%) was observed at 0.8 mg/ml, with an IC_50_ value of (0.41 mg ml). It was difficult to compare this report because this is the first report that indicates the antihypertensive properties of this marine plant, but this positive effect suggests that it could enhance the biological properties of this attractive ingredient for future application in the remedy for hypertension.

## Conclusion

This work had as objectives the characterization and the contribution to the valuation of the *C. nodosa* seagrass based on the lack of results on its techno-functional and biological properties in Tunisia.

The nutritional value of this marine plant derives from the fact that they are characterized by an important composition in dietary fiber, minerals with relatively high levels of protein and lipids and some polyunsaturated fatty acids. Indeed, the relative results of chemical analysis, morphology, texture, particle comportment and functional properties highlight his optimal usage levels in foods since a desirable texture should be retained. In fact, the wealth, especially in dietary fiber has the ability to limit the occurrence of certain diseases (hypertension) with the oxidative stress damages.
